# Effects of Cord Serum Insulin, IGF-II, IGFBP-2, IL-6 and Cortisol Concentrations on Human Birth Weight and Length: Pilot Study

**DOI:** 10.1371/journal.pone.0029562

**Published:** 2011-12-29

**Authors:** Arianna Smerieri, Maddalena Petraroli, Maria Angela Ziveri, Cecilia Volta, Sergio Bernasconi, Maria E. Street

**Affiliations:** Department of Paediatrics, University Hospital of Parma, Parma, Italy; John Hopkins Bloomberg School of Public Health, United States of America

## Abstract

**Background:**

The IGF system is recognised to be important for fetal growth. We previously described increased Insulin-like growth factor binding protein (IGFBP)-2 cord serum concentrations in intra-uterine growth retardation (IUGR) compared with appropriate for gestational age (AGA) newborns, and a positive relationship of IGFBP-2 with Interleukin (IL)-6. The role of cortisol in the fetus at birth is largely unknown, and interactions among peptides are their real effect on birth size is unknown. Furthermore, almost all studies have previously assayed peptides in serum several years after birth, and follow-up data from pregnancy are always lacking. This study aimed at establishing and clarifying the effect of cord serum insulin, IGF-II, IGFBP-2, cortisol and IL-6 concentrations on birth length and weight.

**Methods:**

23 IUGR and 37 AGA subjects were followed up from the beginning of pregnancy, and were of comparable gestational age. Insulin, IGF-II, IGFBP-2, cortisol and IL-6 concentrations were assayed in cord serum at birth, and a multiple regression model was designed and applied to assess which were the significant biochemical determinants of birth size.

**Results:**

Insulin, cortisol, and IL-6, showed similar concentrations in IUGR and AGA as previously described, whereas IGF-II was lower, and IGFBP-2 increased in IUGR compared with AGA. IGF-II serum concentration was found to have a significant positive effect on both birth length (r:^:^0.546; p: 0.001) and weight (r:0.679; p: 0.0001). IGFBP-2 had a near significant negative effect on both birth weight (r:−0.342; p: 0.05) and length (r:−0.372; p:0.03).

**Conclusion:**

IGF-II cord serum concentration was shown to have a significant positive effect on both birth length and weight, whereas IGFBP-2 had a significant negative effect. Insulin, cortisol, and IL-6 cord serum concentrations had no significant effect on birth size.

## Introduction

Fetal growth is driven mainly by the IGF system as experiments in knockout mice have shown [1], [2]. IGF-II and the type I IGF receptor are thought to be the most important for fetal growth [3], [4].

We described increased IGFBP-2 cord serum concentrations in IUGR compared with appropriate for gestational age (AGA) newborns, and a positive relationship of serum IL-6 with IGFBP-2 [5], although IL-6 concentration did not show any changes in the two conditions. We described instead increased IL-6 mRNA and protein concentrations in placental lysates from IUGR [5], and our recent evidence suggested a negative effect, although not a major effect, of IL-6 placental concentration on birth size (our unpublished data).

Very little data has been published on the fetus to date. Certainly it is of interest to consider the described molecular mechanisms by which IL-6 can induce insulin-resistance [6], [7].

Furthermore, animal and human studies showed a marked activation of the hypothalamic-pituitary-adrenal axis by IL-6 infusion [8]–[10].

We previously described similar cortisol and insulin concentrations in cord serum of newborns, both IUGR, and AGA.

The role of cortisol in the fetus at birth is largely unknown. Interestingly, Cianfarani et al. reported a positive correlation of cortisol serum concentrations with IGFBP-1, and a negative correlation with IGF-I and length gain in the first 3 months of life, in normal neonates [11]. The same authors subsequently reported higher cortisol serum concentrations at the age of nine years in IUGR subjects who failed to catch-up with growth, compared with those who caught-up, and a negative relationship with birth weight. It was also suggested that the increased cortisol serum concentrations reduced IGFBP-3 proteolysis reducing IGF bioavailability [12].

IUGR and appropriate for gestational age (AGA) newborns show differences but are subjected overall to the same biological laws.

We followed-up a unique series of newborns, both IUGR and AGA, from pregnancy to birth [5], [6], and assessed in cord serum which peptides had a significant effect on birth length and weight, among insulin, IGF-II, IGFBP-2, cortisol and IL-6.

## Materials and Methods

### Subjects

Twenty-three IUGR (13M, 10F) and 37 AGA (18M, 19F), births of comparable gestational age (34.8±0.6 vs. 36.1±0.4 weeks, respectively, n.s.) were followed during pregnancy. All pregnancies were dated correctly by ultrasound during the first trimester of gestation.

AGA newborns were defined on the basis of a normal birth weight (<80th and >10th centiles) with respect to the Italian standards [13], a normal pregnancy and the absence of maternal risk factors.

The diagnosis of IUGR was made within the 32nd week of gestation and was ascribed to a probable placental cause after excluding other causes as infections, chromosomal abnormalities, genetic syndromes, maternal malnutrition, substance abuse including smoking, gross placental abnormalities and multiple fetuses. No cases with hypertension, gestational diabetes or reduced amount of amniotic fluid were included in the study. Mothers were all normal-weight subjects and data on parity, were collected also.

The IUGR pregnancies were diagnosed by ultrasound according to the following criteria: abdominal circumference <10th centile and/or shift of fetal growth with a reduction of abdominal circumference with respect to the measure taken during the 20th week of gestation. In IUGR subjects, doppler velocimetry was altered in almost all cases in the placenta and/or fetus site. All neonates, both IUGR and controls, were delivered by elective caesarean section (CS).

The causes of preterm birth (<38 weeks of gestation) in the AGA newborns were: intra-hepatic cholestasis in a woman who had underwent a previous CS, premature rupture of membranes with breech presentation and delivery within 4 hours with no signs of infection. Elective CS in these subjects was performed because of refusal of vaginal delivery for psychological reasons, or because of a previous CS.

We could obtain a complete data-set on which this study was performed in 34 subjects. The main clinical features of these newborns are shown in Table 1.

**Table 1:** Main clinical features at birth of both IUGR and AGA newborns.

	IUGR (13: M6, F7 )	AGA (21: M9, F12 )	p-Value
Gestational age (yr)	35.7±0.5	36.9±0.4	n.s.
Mother's chronological age (yr)	32.0±1.2	34.2±0.7	n.s.
Mother's BMI at beginning of pregnancy (kg/m2)	22.6±0.9	22.3±0.7	n.s.
Mother's birth weight (kg)	3.2±0.2	3.4±0.1	n.s.
Father's birth weight (kg)	3.2±0.2	3.4±0.1	n.s.
Parity	1.86±0.4	2.25±0.2	n.s.
Placental weight (g)	341.7±31.6	601.0±21.8	0.0001
Birth weight (kg)	1.8±0.3	2.9±0.1	0.001
Birth length (cm)	41.1±1.1	49.9±0.5	0.0001
Birth head circumference (cm)	29.2±0.7	34.4±0.4	0.0001

Data are mean ± SEM. The comparison between the 2 groups was done using an unpaired T-test.

### Data collection and measuring techniques

Length (Lt) and weight (Wt) at birth was measured by trained midwives at our hospital using exact measuring equipment for length measurements. At birth auxological data were plotted according on to the Italian standards [13].

At birth the following information was also collected: age of the mother, weight at birth of both parents, body mass index (BMI) of the mother before pregnancy, previous gynaecological history, medical history during pregnancy, fetal biophysical data (exact duration of pregnancy, growth trend), clinical data at delivery (indication for CS, neonatal data as sex, weight, length, head circumference, APGAR score, acid-base equilibrium, perinatal data), weight and macroscopic aspect of the placenta.

### Collection of biological material

Umbilical cord serum from IUGR and AGA births were collected at birth and treated as previously described [5]. The blood was delivered to the laboratory within 20 minutes, centrifuged (2000 g/min for 10 minutes at 4°C) and the sera aliquoted and stored at −80°C until assayed.

### Cord serum assays

Peptides were assayed as previously described, prior to this study [5], [6].

Briefly, IGF-II was measured using an IRMA method (Diagnostic System Laboratories, Inc. Webster, Texas, USA).

IGFBP-2 was assayed using a RIA method (Diagnostic System Laboratories, Inc. Webster, Texas, USA).

Insulin was assayed using a specific IRMA assay (DiaSorin S.p.A., Saluggia, Italy).

IL-6 was measured using an ultrasensitive ELISA method (Quantikine HS, R&D Systems, Minneapolis, MN, USA).

Cortisol was assayed using a specific RIA assay (DiaSorin S.p.A., Saluggia, Italy).

### Ethical approval

Informed consent was obtained from the mothers. The study was approved by the Ethics Committee of the University of Parma Medical School.

### Statistical analysis

Statistical analysis was performed using SPSS 18.0 for Windows (SPSS, Inc, Chicago, IL).

A complete data-set with both growth parameters at birth, and all peptide concentrations in placenta could be obtained for 34 subjects.

Results for males and females were combined as in this series we did not detect any significant difference between the two sexes.

Prior to the study we compared concentrations between cord serum peptide concentrations to check whether previous reported differences were confirmed in this population using an unpaired T test. Results are reported in Table 2, and confirmed our previous findings [5], [6].

**Table 2:** Concentrations of cord serum peptides in IUGR and AGA at birth.

	IUGR	AGA	p-Value
Insulin (pmol/ml)	55.2±13.8	70.3±4.9	n.s.
IGF-II (ng/ml)	461.9±21.6	646.7±26.7	0.017
IGFBP-2 (ng/ml)	2685.8±430.6	1175.8±144.7	0.006
Cortisol (pmol/ml)	295.2±33.1	275.9±13.8	n.s.
IL-6 (pg/ml)	4.7±1.8	3.2±0.5	n.s.

Data are mean ± SEM. Comparisons between the two groups were done using an unpaired T-test.

The normal distribution of the data was determined using the Kolmogorov-Smirnov test.

Multiple linear regression analysis was used, with stepwise eliminations of the independent variables, to study the effect of individual peptides on parameters of birth size of both IUGR and AGA newborns, as both populations are subjected to the same general growth rules, and to obtain acceptable statistical power. No significant effect of gestational age was found as expected, considering that gestational ages were similar.

Parity was ruled out also as was similar in the two groups.

We used Bonferroni correction to correct for multiple linear regression modeling. Statistical significance was set at a p-value less than 0.007 (p: 0.05/x; x = 7).

For the analysis of the variables influencing both length and weight at birth, the independent variables were insulin, IGF-II, IGFBP-2, cortisol, and IL-6 concentrations in cord serum at birth.

## Results

### Determinants of birth length

In the multiple regression model the overall R^2^ was 0.570 (p:0.0001).

IGF-II cord serum concentration was shown to have a significant positive effect on birth length (p: 0.001) (Table 3).

**Table 3:** Effect of cord serum insulin, IGF-II, IGFBP-2, cortisol, and IL-6 on birth length.

	B	Partial Correlation	p-VALUE*
Insulin (pmol/ml)	0.126	0.190	0.298
IGF-II(ng/ml)	0.523	0.546	0.001
IGFBP-2(ng/ml)	−0.321	−0.372	0.030
Cortisol (pmol/ml)	0.068	0.580	0.580
IL-6 (pg/ml)	−0.195	−0.282	0.118

*p-values were corrected using Bonferroni's criteria.

IGFBP-2 cord serum concentration had a negative effect on birth length (p:0.03) (Table 3).

No significant effect of insulin, cortisol, and IL-6 was shown.

### Determinants of birth weight

In the multiple regression model the overall R^2^ was 0.461 (p:0.0001).

IGF-II cord serum concentration was shown to have a significant positive effect on birth weight ( p: 0.0001) (Table 4).

**Table 4:** Effect of cord serum insulin, IGF-II, IGFBP-2, cortisol, and IL-6 on birth weight.

	B	Partial Correlation	p-VALUE*
Insulin (pmol/ml)	0.171	0.230	0.198
IGF-II (ng/ml)	0.679	0.679	0.0001
IGFBP-2 (ng/ml)	−0.307	−0.342	0.050
Cortisol (pmol/ml)	0.021	0.029	0.874
IL-6 (pg/ml)	−0.210	−0.286	0.107

*p-values were corrected using Bonferroni's criteria.

IGFBP-2 cord serum concentration had a negative effect on birth weight (p:0.05) (Table 4).

## Discussion

Among the cord serum peptides analysed, we described IGF-II and IGFBP-2 as the peptides having the most important effect on both birth length and weight. In this pilot study, insulin, cortisol, and IL-6 cord serum concentrations did not seem to have a major effect on birth size.

Data in the Literature to date, concerning the relationship of IGF-II with fetal growth are still controversial [14]–[18]. Our IGF-II finding is in accordance with the experimental evidence in animal models, in particular, in mouse models [3], [19]–[20]. Recent data have further proven, from a genetic point of view, the growth-promoting function of IGF-II during mouse embryogenesis, which has been shown to be mediated in part by signalling through the insulin receptor [19].

Interestingly, IGF-II has been shown to have also a critical role for neural and cardiovascular development in zebrafish embryos [21], and in general distinct functions have been proven during organogenesis, including the embryonic development of the kidneys [22].

Our finding is in agreement with previous studies also, in humans, showing that IGF-II gene polymorphisms are associated with birth weight and fetal growth, as some gene polymorphisms through IGF-II gene expression clearly associate with birth weight SDS [23], [24]. However, there are few and controversial data on the correspondence of gene polymorphisms and IGF-II serum concentrations [24]. Finally, studies have shown that IGF-II is also capable of stimulating somatic overgrowth in utero [25].

We previously described increased IGFBP-2 cord serum concentrations in IUGR compared with AGA newborns, however, the biological meaning of that finding with respect to other changes detected in serum in IUGR at that time was unknown, as well as clear relationships with birth length and/or weight [5].

This pilot study now suggests a major negative effect of IGFBP-2 on both birth length and weight, altogether a novel and interesting finding, in particular, in the light of recent publications. IGFBP-2 has been found in fact to be possibly the most useful, among other peptides of the IGF system, in assessing nutritional status in preterm neonates during the early postnatal period [26], and in adults a strong association between low IGFBP-2 levels and metabolic syndrome has been identified [27]. A recent study has further shown that during clamp studies, insulin increases IGFBP-2, and that IGFBP-2 was an independent predictor of insulin sensitivity. This suggested that this peptide played an important role in the insulin-IGF cross talk and was thus closely linked to insulin resistance [28]. Moreover, in obesity, IGFBP-2 was shown recently to reflect long-term insulin sensitivity [29].

Interestingly, in recent years independent effects of IGFBP-2 on glucose metabolism have been discovered also [30]. A recent report in young adults born small for gestational age suggested that IGFBP-2 could be a cardiovascular risk marker in later life [31]. However, these findings in adults are all related to serum concentrations in later life compared to our subjects, and it is yet unknown whether cord serum concentrations could anticipate later findings.

Altogether, our findings related to IGF-II and IGFBP-2 still suggest an important role of insulin, although our analyses suggested an indirect and not a major effect. Cortisol and IL-6 in the fetus at birth, do not seem to have a major role in determining birth size, however, the model did not exclude completely an effect which could also be indirect. Interestingly, our recent data show a positive effect of insulin placental concentration on both birth length and weight (unpublished), although the major effect would be mediated through cortisol placental concentration.

In conclusion, the findings of this pilot study evidenced a relationship, in particular, of IGF-II and IGFBP-2 cord serum concentrations with birth size, and also could suggest a possible relationship of fetal size with insulin sensitivity in later life, as previous clinical observations have hypothesized [32].
